# Inkjet Printing of Super Yellow: Ink Formulation, Film Optimization, OLEDs Fabrication, and Transient Electroluminescence

**DOI:** 10.1038/s41598-019-44824-w

**Published:** 2019-06-11

**Authors:** Amruth C, Marek Zdzislaw Szymański, Beata Łuszczyńska, Jacek Ulański

**Affiliations:** 1 Department of Molecular Physics, Lodz University of Technology, 90-924 Lodz, Poland; 2 Department of Engineering and Chemical Sciences, Karlstad University, SE-651 88 Karlstad, Sweden; 3 School of Science and Technology, Örebro University, SE-701 82 Örebro, Sweden

**Keywords:** Electronic devices, Design, synthesis and processing, Organic LEDs

## Abstract

Inkjet printing technique allows manufacturing low cost organic light emitting diodes (OLEDs) in ambient conditions. The above approach enables upscaling of the OLEDs fabrication process which, as a result, would become faster than conventionally used vacuum based processing techniques. In this work, we use the inkjet printing technique to investigate the formation of thin active layers of well-known light emitting polymer material: Super Yellow (poly(para-phenylene vinylene) copolymer). We develop the formulation of Super Yellow ink, containing non-chlorinated solvents and allowing stable jetting. Optimization of ink composition and printing resolution were performed, until good quality films suitable for OLEDs were obtained. Fabricated OLEDs have shown a remarkable characteristics of performance, similar to the OLEDs fabricated by means of spin coating technique. We checked that, the values of mobility of the charge carriers in the printed films, measured by transient electroluminescence, are similar to the values of mobility measured in spin coated films. Our contribution provides a complete framework for inkjet printing of high quality Super Yellow films for OLEDs. The description of this method can be used to obtain efficient printed OLEDs both in academic and in industrial settings.

## Introduction

The organic light emitting diodes (OLEDs) technology has been developed for applications such as flat panel displays, flexible displays and solid state lighting. OLED based displays, compared to liquid crystal displays (LCDs), have significant advantages, such as higher viewing angles, higher contrast, lower weight and faster response^1–3^. OLEDs currently available on the market are manufactured using high cost vacuum evaporation technology, which makes the end products expensive^4^. Therefore, the use of an alternative fabrication process is highly desirable to reduce the manufacturing cost. In this respect, printing techniques attract much attention because of their lower fabrication cost, especially for large area devices. Printing techniques allow for manufacturing of large area devices on flexible substrates through roll-to-roll process, which enables high volume production^5^. Printing techniques include, for example, gravure^6^, screen^7^, flexographic^8^, and inkjet printing^9^. Among these, inkjet printing is extensively used to fabricate OLEDs^10^. It is one of the most promising methods of industrial production of OLEDs, which has advantages of comparatively high-speed of fabrication, low material consumption and suitability for mass production^11^. Since this technique does not use mask, it allows depositing the active layer via non-contact process. As a result, the substrate remains free from contamination during fabrication process. Furthermore, this technique may be better controlled as it allows for generating the drops on demand and facilitates selective patterning^12,13^.

Notwithstanding the many advantages of inkjet printing technique, there are still many challenges to overcome in order to print good quality films which would be used in an OLED structure. These challenges include the formulation of appropriate inks which could produce spherical drops and show a stable jetting^14^. It should be noted that the use of halogenated solvents for ink formulation is not preferred for industrial applications, because they can cause serious health and environmental risks^15^. Furthermore, after inkjet printing on a substrate, problems such as “coffee ring effect” can give rise to the formation of non-uniform films^16^. Therefore, it is necessary to optimize the ink and the printing parameters to avoid such undesirable effects. Thus, for each layer of the device, it is necessary to optimize the ink in respect of formulation, film formation-ability, and performance in the device.

Conjugated polymers are extensively used in OLEDs due to their tunable optoelectronic properties, flexibility and good solubility in organic solvents. Poly(para-phenylene vinylene) copolymer known as Super Yellow (SY) is one of the most widely used conjugated polymers for solution processable OLEDs^17–20^. Interestingly, Super Yellow has similar mobility properties for both holes and electrons. Such a balanced ambipolar transport is a crucial property required for obtaining efficient OLEDs^21^, while in most conjugated polymers the hole mobility is usually higher than the electron mobility. Nonetheless, it should be noted, that most of literature reports focus on Super Yellow thin films prepared by spin coating technique. These reports and results are not directly applicable for optimization of printing process. Also, to our best knowledge, there are no comprehensive reports on OLEDs with inkjet printed Super Yellow.

In this contribution, we would like to present a method of fabrication of Super Yellow thin films by means of inkjet printing technique. This includes ink formulation and the tuning of the printing process in order to obtain stable drop formation. The subject of our examination includes the influence of Super Yellow concentrations, the printing resolution, and the substrate temperature on the film formation. The printed films are described by measuring the thickness, and roughness, and by photoluminescence spectroscopy. Additional thin SY films were made by spin coating technique, and used as a reference point for these fabricated by inkjet printing. After developing inkjet printing procedure yielding high quality SY films, we fabricated OLEDs with varying thickness of the active layer, in order to optimize the active layer thickness. Finally, we used transient electroluminescence technique in order to describe charge carrier transport properties of the fabricated films.

## Results and Discussion

### Influence of solvent and polymer concentration on inkjet printing

Toluene is the most common solvent used for preparation of Super Yellow solutions^19,20,22^. Therefore, we started our investigation by preparing inks with different concentrations of Super Yellow dissolved in toluene. We discovered that the inks with concentration of Super Yellow exceeding 0.5 mg/mL are non-jettable. Most probable reason was the nozzle clogging, caused by the rapid evaporation of the solvent at the nozzle-air interface^23^. Hence, 0.5 mg/mL (SY INK-1) is the maximum printable ink concentration. When used, printed films show non-homogeneous surface, regardless of printing resolution. An example of optical image of printed film at printing resolution of 900 dpi is shown in the Supplementary Fig. S3. The film had low thickness of 10 nm, with large variation of 10 nm. Such a film is too thin and too non-uniform for the OLED applications. We attribute this to the insufficient concentration of the solute, and the fast drying rate of the film. The latter prevents merging of drops, especially in the orthogonal direction of printing. Thus, to obtain thicker and more uniform films suitable for OLEDs, it is necessary to increase the concentration of Super Yellow, and to decrease the drying rate.

It is known that an improvement of ink properties is possible by mixing low boiling point solvent (main solvent) with higher boiling point solvent (co-solvent)^24^. This often permits to avoid clogging of the nozzle, because the solvent with higher boiling point keeps the nozzle wet. Furthermore, the use of such a solvent mixture decreases the drying rate of the film. The decreased drying rate gives sufficient time for the spreading of the ink within the film area, and as a result it helps to avoid non-uniform films^25,26^. In our study, we use tetralin as a non-chlorinated high boiling point co-solvent. Toluene and tetralin in 75:25 volume ratio are used for the ink composition. Using this solvent mixture, we tested inks with different concentrations of Super Yellow and we found that concentrations below 3 mg/mL will give best results for inkjet printing. All following results were obtained by means of this ink formulation with concentrations of either 2 mg/mL (SY INK-2) or 3 mg/mL (SY INK-3), unless stated otherwise.

### Formulation of ink

Physical properties of the ink, such as the surface tension and the viscosity, play an important role in the drop formation process^23^. Formm^27^ derived a dimensionless number Z which consider the physical properties of an ink*, Z* = (*aγρ)*^*1/2*^*η*^*−1*^, where, *a* is the radius of the nozzle, *γ* is the surface tension, ρ is the density of the fluid, and *η* is the viscosity. The *Z* number allows to predict whether a liquid can be used as ink for inkjet printing. Formm^27^ predicted that, when *Z* > 2, liquids can be printable. Furthermore, Jang *et al*.^28^ determined that liquids characterized by *Z* values in the range from 4 to 14 are usable as inks for inkjet printing.

The surface tension, the viscosity and the density of the SY INK-2 ink were measured as: 7 cP, 30 dyne/cm and 0.988 g/cm^3^ respectively. The surface tension, the viscosity and the density of the SY INK-3 were measured as: 9.1 cP, 30 dyne/cm and 0.988 g/cm^3^ respectively. For the nozzle with radius 35 µm, the *Z* value of SY INK-1 and SY INK-2 are, respectively, 4.2 and 3.5, which indicates that the inks are in printable range (see Supplementary Table S1).

**Figure 1:**
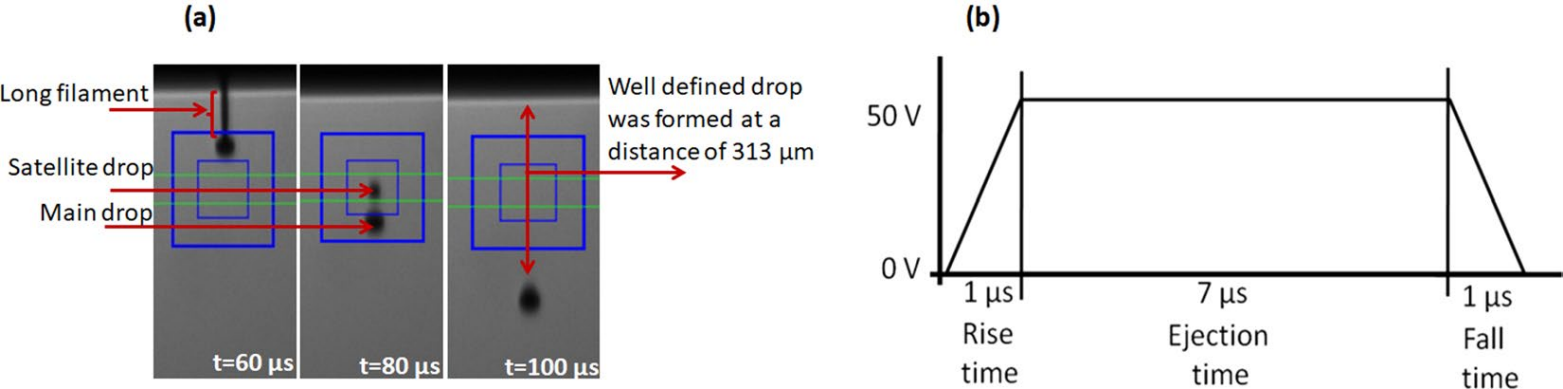
(**a**) Sequential image of drop formation of the SY INK-3 ink taken at three different values of time elapsed after ejection from piezo-head; (**b**) Waveform used to drive the piezo-head.

Figure 1a shows the images of drop formation of SY INK-3 taken at different times after the ejection from LP 50 inkjet printer (see experimental details). After 60 µs, the drop is still connected with the nozzle by a filament. At a time of 80 µs the drop detaches from the nozzle, and the filament forms a satellite drop which follows the main drop. After 100 µs, a well-defined spherical drop is obtained, which is free from a satellite drop or a filament. The velocity and the volume of the drop at 100 µs after ejection are: 3.13 m/s and 15 pL respectively. The deviation angle of the drop from the nozzle is 2.15°. Figure 1b shows the driving waveform, which is optimized to obtain a spherical drop. This voltage signal is delivered to the piezo-head to eject each drop. Using the time required for the formation of spherical drop and the determined velocity of the drop, it is possible to determine the minimum distance from the nozzle at which the drops become spherical, and it is approximately 300 µm. The distance between the substrate and the printhead during the printing process was set to be fixed at 1 mm. Since we are able to obtain spherical drops at a distance of approximately 300 µm, it is evident that the spherical drops will arrive at the surface of the substrate.

### Interaction between Ink and substrate

**Figure 2:**
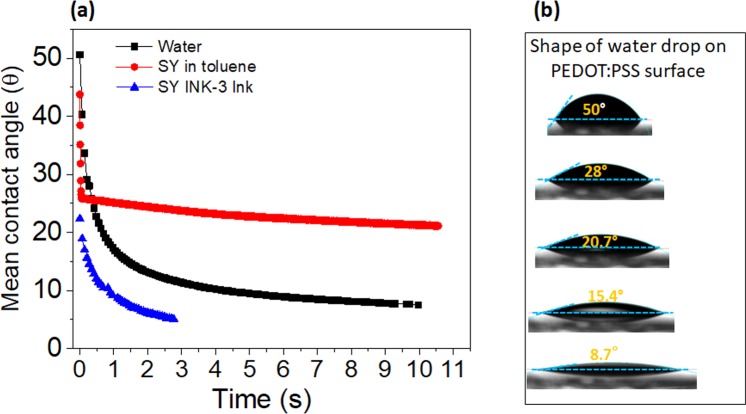
(**a**) Contact angle of water and two types of SY ink on PEDOT:PSS surface. The volumes of drops are approximately 4 µl. Contact angle shown here is the average of right and left contact angles (**b**) Shape of the drop of water at different contact angles.

The wettability of printed surface plays a crucial role in the formation of uniform and continuous films^29^. In order to investigate the wettability of the PEDOT:PSS surface, we have measured the dynamic contact angle of water, and of SY INK-3 (see in Fig. 2a). The shape of water drop at various contact angles is presented in Fig. 2b. Water spreads quickly (in 5 to 10 sec) on the PEDOT:PSS, with the contact angle less than 10°, which indicates complete wetting behavior. This result suggests that the surface is highly hydrophilic. SY INK-1 shows contact angle of approximately 22°, which indicates a good wetting behavior of this solvent. A drop of SY INK-3 spreads immediately with contact angle less than 5°. This shows excellent wetting behavior of the optimized ink, which can be printed on PEDOT:PSS without surface treatment.

### Influence of print resolution on film formation process

**Figure 3:**
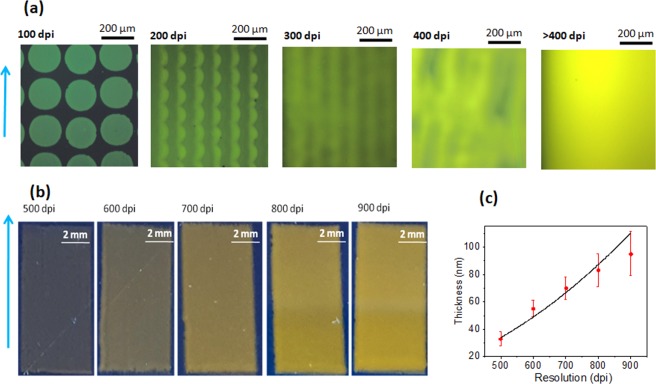
Films printed with SY INK-3 at various printing resolutions on a spin coated PEDOT:PSS surface. (**a**) Optical microscopic images under UV illumination. Blue arrow indicates printing direction. (**b**) Pictures of printed films taken from a digital camera. The starting point of printing is at left bottom corner and the printing direction is shown with a blue arrow. Pictures of films printed with a resolution below 400 dpi were not presented as they were too transparent to capture. (**c**) Thickness as a function of printing resolution.

We investigated the influence of resolution on film formation, in the range of resolution from 100 dpi to 900 dpi. Films printed with low resolutions, between 100 dpi and 400 dpi, are discontinuous. Using resolutions above 400 dpi allows to obtain uniform films (Fig. 3a).

At a low resolution of 100 dpi, individual drops with ~20 µm diameter are obtained. When the resolution was increased to 200 dpi, drops started to connect together to form continuous films. At low printing resolutions the films have anisotropic surface, with clearly visible printing direction (see in Fig. 3a). The anisotropy disappears at a printing resolution of over 400 dpi. Figure 3b shows pictures films printed with resolutions from 500 dpi to 900 dpi. Assuming that there is no migration effect of the materials to the edges of the printing area, known as “coffee ring effect”, the film thickness should scale with second power of the resolution. In most cases the coffee ring effect is stronger at a higher resolution so we can observe some deviations from theoretical behavior, as it is seen in Fig. 3c.

**Figure 4:**
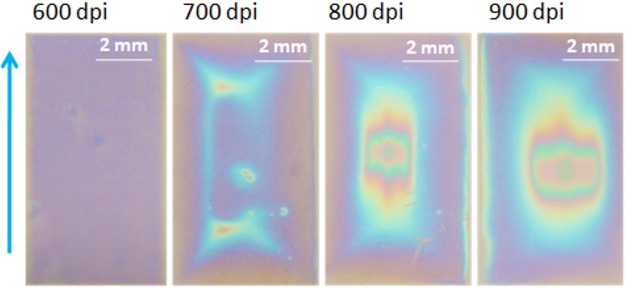
Pictures (taken with a digital camera) of films printed with SY INK-2 on a spin coated PEDOT:PSS with different printing resolutions. Blue arrow denotes the direction of printing.

Figure 4 shows images of inkjet printed films obtained from a solution with SY INK-2. Best films were printed with a resolution of 600 dpi, which resulted in the uniform thickness of 20 ± 5 nm. Films printed with a higher or a lower value of resolution showed worse thickness uniformity.

### Influence of substrate temperature on inkjet printed film formation

The Super Yellow film with SY INK-3 was printed at various substrate temperatures: 23 °C (ambient temperature), 30 °C, 35 °C, 40 °C, 50 °C and 60 °C. The print resolution was kept at 700 dpi.

Super Yellow films printed on the substrates both at ambient temperature and at 30 °C were uniform and homogeneous (see Supplementary Fig. S4). Under increase of substrate temperature to 35 °C and higher, the printed films show non-uniformity. Films printed on the substrates at temperatures respectively 35 °C and 40 °C had many voids across the film. Films printed on the substrates at temperatures of 50 °C and 60 °C were characterized by line like structure in the printing direction. The appearance of defects with increased substrate temperature is caused by the increase in the evaporation rate of the solvents. It has been already reported that, at higher temperatures of printed substrate, the solvents start evaporating already during the time of jetting of the ink. As a consequence, the drop diameter is decreased at the time of hitting the substrate. The smaller diameter of drops, and smaller content of solvent, prevents the coalescence of the drops, resulting in the inhomogeneity in the printed films^24^. The effect was further investigated by Arjun *et al*.^30^, reporting that, under an increase of substrate temperature, the contact angle increases as the evaporation rate increases. Both effects restrict the spreading of the ink. In our case, the substrate temperature below 30 °C was optimal to obtain uniform films.

### Surface roughness of printed film

The film was printed using the SY INK-3. The inkjet printed film is characterized by a root mean square roughness (R_q_), average roughness (R_a_), and the total height of roughness profile (R_t_) of 0.55 nm, 0.45 nm and 2.31 nm respectively (see Supplementary Fig. S5a–d). These values of roughness are very similar to the respective roughness values of spin coated films. The spin coated film has R_q_, R_a_ and R_t_ of 0.46 nm, 0.37 nm and 1.7 nm, respectively. The measurements of roughness show that the inkjet printed films have a smooth topology similar to the spin coated films.

### Optical spectroscopic study

The study of optical absorption spectra has shown, that peaks of maximum absorption of SY in the solution, and in spin coated or printed films have the same wavelength position of 450 nm. Absorption bands of both the spin coated and the printed films are almost identical (see Supplementary Fig. 6Sa). However, the absorption bands observed for solid-state films are broader than the ones for the solution. This can be attributed to the formation of aggregations in the solid films^31^. The photoluminescence spectra of solution show a prominent peak at 523 nm and a smaller peak at 556 nm. The spin-coated films have corresponding peaks of emission at wavelengths of 555 nm and 571 nm. The inkjet printed films have emission peaks at wavelengths of 549 nm and 566 nm, respectively. The emission spectra of both spin coated and printed films are similar to each other, and the emission band in the solid state is red shifted in comparison to the solution (see Supplementary Fig. 6Sb). This is likely caused by aggregation^32^.

### OLED performances

**Figure 5:**
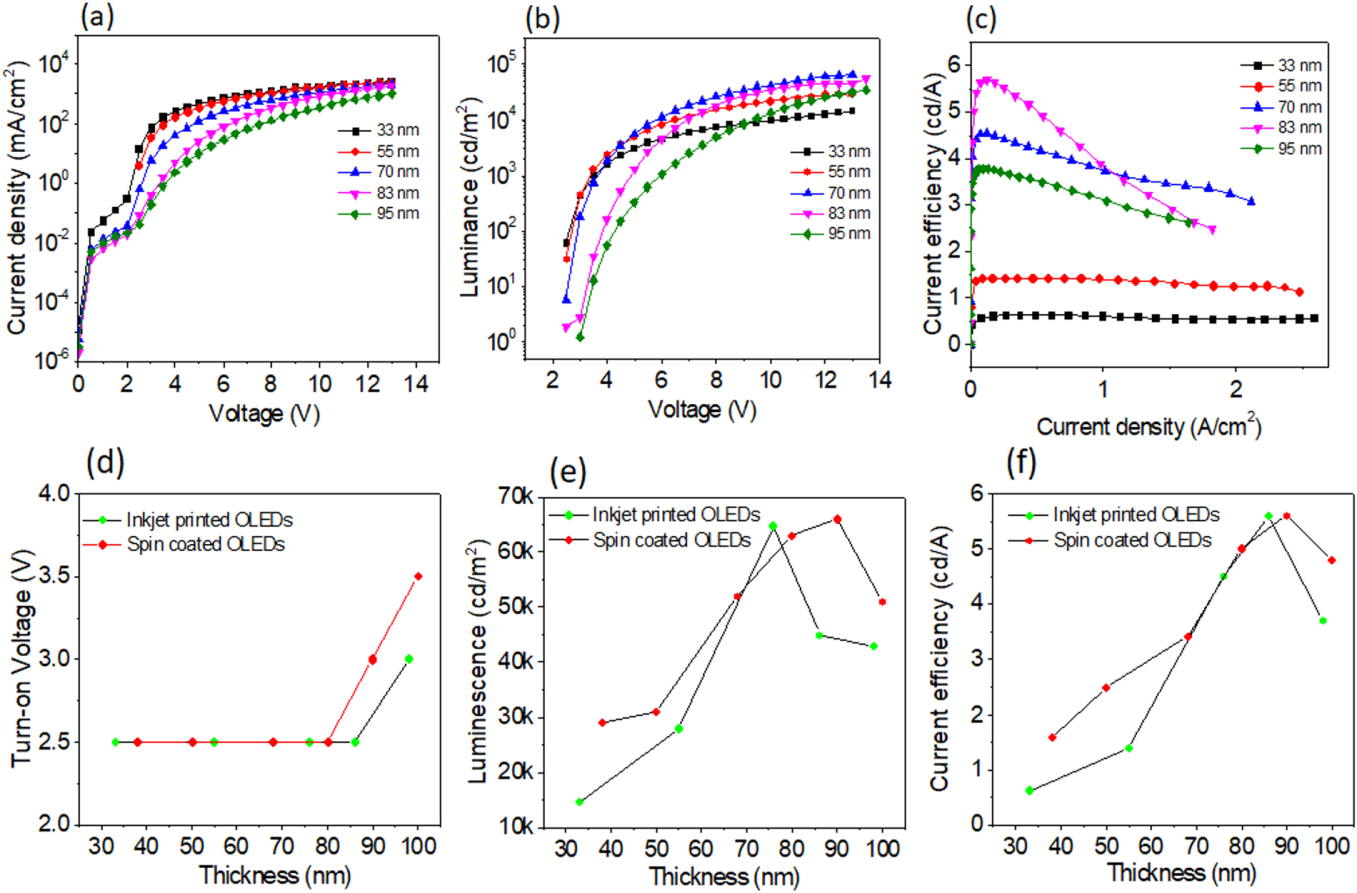
Results of opto-electronic characterization of inkjet printed OLEDs, as a function of thickness of the active layer: (**a**) current density - voltage characteristics (**b**) luminance - voltage characteristics (**c**) current efficiency - current density characteristics; (**d**) turn-on voltage as a function of thickness (**e**) maximum luminescence as a function of thickness (**f**) maximum efficiency as a function of thickness. For reference, values measured for OLEDs fabricated by spin-coating and otherwise identical are given in plots (**d–f**), as red points.

We have printed a series of OLEDs with varying thickness of the active layer using SY INK-3. Different thicknesses were realized by changing the resolution of printing (see Supplementary Table S2; Fig. 3c). Figure 5a–c present, respectively, current density - voltage, luminance - voltage and current efficiency - current density characteristics of printed OLEDs. Turn-on voltage, maximum luminescence, and maximum efficiency of printed OLEDs, as a function of thickness of the active layer, are given in Fig. 5d–f and Supplementary Table S3.

As expected, the current density of the printed OLEDs increases as the thickness of active layer decreases (see in Fig. 5a), due to decreasing resistance of the layer. The turn-on voltage, defined as a voltage at which the luminescence intensity is 1 cd/m^2^, was in the range 2.5–3 V for all OLEDs. The plots of maximum luminance and current efficiency **(**Fig. 5e,f**)** reveal that the best performance is achieved when the thickness of the Super Yellow active film is between 70 nm and 85 nm. The optimal thickness is determined by overall device physics of OLEDs^33–35^, including light out-coupling effects^18^, and its discussion is beyond the scope of this paper.

For reference, we fabricated OLEDs identical to the inkjet printed devices, by spin coating. The thickness of spin coated Super Yellow films was controlled by the spin speed (see Supplementary Table S4). Full characteristics of spin coated devices are shown in the Supplementary Fig. S7, Supplementary Table S5. Comparisons between the performance of spin-coated OLEDs and the printed OLEDs are presented in the Fig. 5d–f. As shown, the performances of inkjet printed and spin-coated OLEDs are almost identical. The differences could be partially explained by slightly different transport properties of active layers (Fig. 7).

**Figure 6:**
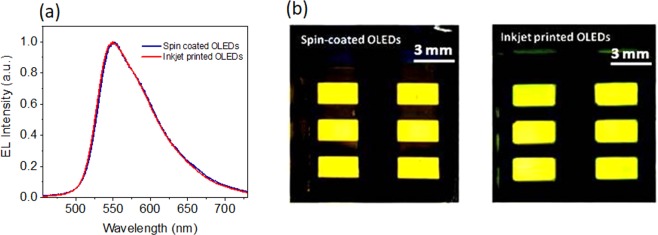
(**a**) Electroluminescence spectra of a spin coated OLED and a printed OLED, with a thickness of 85 nm and an applied of voltage 7 V. (**b**) Uniform emission of light from printed and spin coated OLEDs under voltage bias.

Figure 6a shows comparison of electroluminescence spectra (EL) of a spin coated OLED and an inkjet printed OLED, measured at applied voltage 7 V. The thickness of active layer was 85 nm in each case. Both electroluminescence spectra are close to identical. This shows that the use of inkjet printing technique for the deposition of Super Yellow films has no effect on the EL emission spectrum. Furthermore, the emission from the inkjet printed OLEDs is spatially homogeneous, just as the emission of the spin coated OLEDs (Fig. 6b).

### Transient electroluminescence

Mobility of charge carriers (µ) is one of the most important parameters determining the transport properties of semiconducting thin films^36^. Transient electroluminescence (TEL) measurement technique is widely used to measure the charge carrier mobility in active layers of OLEDs^37–40^. With this technique one can obtain the mobility of majority charge carriers in a single carrier film or an effective mobility of the charge carriers in a double carrier film. In the latter case, the effective mobility is understood as the sum of the effective hole mobility (µ_h_) and the effective electron mobility (µ_e_)^41^.

This technique involves application of a voltage pulse to the device, and observing the time delay in the electroluminescence signal. On the application of a voltage pulse, carriers are injected from the respective electrodes, and start to travel towards opposite electrodes. Opposite charge carriers meet after a certain time, and recombine emitting photons. As a result, there will be a delay time (t_d_) between the rising edge of the applied voltage and the onset of luminescence. By knowing the delay time, the effective mobility of the charge carriers can be calculated from the below Eq. (1) ^42^.

1 $${\mu }_{eff}=\frac{{d}^{2}}{{t}_{d}\cdot (V-{V}_{bi})}$$

Where, *µ*_*eff*_ is the effective mobility, *d* is the thickness of the active layer, *t*_*d*_ is the delay time, *V*_*bi*_ is the built-in voltage, *V* is the applied voltage. Here, *V*_*bi*_ is 2.2 V which is calculated as the difference between the work functions of the anode and the cathode.

**Figure 7:**
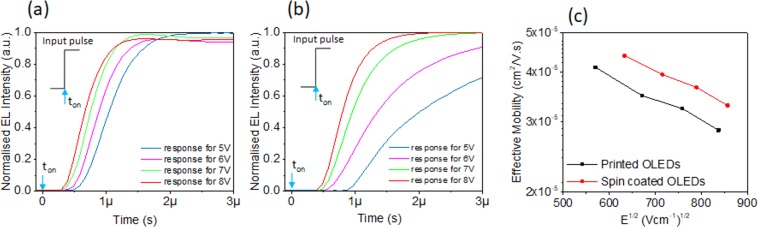
Electroluminescence transient for various input voltage pulse (**a**) inkjet printed OLEDS (**b**) spin coated OLEDs. t_on_ is the rising edge of the input pulse. The thickness of Super Yellow in printed OLED is 83 nm and the thickness of Super Yellow in spin coated OLED is 90 nm. (**c**) Electric filed dependence of effective charge carrier mobility in spin coated and inkjet printed Super Yellow thin film.

Figure 7 shows the normalized input signal and ETL signal for both the inkjet printed OLED **(7a)** and the spin coated OLED **(7b)**. It can be clearly seen that the onset of ETL is delayed with respect to the rising edge of the input voltage pulse. The delay time decreases with the increase in voltage, which is in an agreement with the Eq. (1). The delay times for inkjet printed OLEDs is in the range between 0.4 µs and 0.6 µs and for spin coated OLEDs is in the range between 0.4 µs and 1.08 µs. The effective mobility is calculated using Eq. (1), as presented in Table 1.

**Table 1:** Comparison of the delay time and the effective mobility (µ_eff_), in function of applied voltage, between the inkjet printed and the spin coated OLEDs. The thickness of Super Yellow in inkjet printed OLED is 83 nm and the thickness of Super Yellow in spin coated OLED is 90 nm.

Applied Voltage (V)	Inkjet Printed OLEDs		Applied Voltage (V)	Spin Coated OLEDs	
	(t_D_) Delay Time (µs)	µ_eff_ (cm^2^V^*−*1^s^*−*1^)		(t_D_) Delay Time (µs)	µ_eff_ (cm^2^V^*−*1^s^*−*1^)
5	0.60	4.1 × 10^*−*5^	5	1.08	4.3 × 10^*−*5^
6	0.51	3.4 × 10^*−*5^	6	0.56	3.9 × 10^*−*5^
7	0.43	3.2 × 10^*−*5^	7	0.47	3.6 × 10^*−*5^
8	0.40	2.8 × 10^*−*5^	8	0.41	3.2 × 10^*−*5^

In each case, the effective mobility reported in Table 1 is in the order of 10^*−*5^ cm^2^ V^*−*1^s^*−*1^. This may be seem in disagreement with previous measurements of Tseng *et al*., where reported electron and hole mobilities in Super Yellow are 4.1 × 10^*−*7^ cm^2^ V^*−*1^ s^*−*1^ and 4.1 × 10^*−*7^ cm^2^ V^*−*1^ s^*−*1^ respectively^21^. However, these low values are derived from current- voltage characteristics of single carrier devices. Moreover, Mu *et al*. received the value of hole mobility of 1.6 × 10^*−*7^ cm^2^ V^*−*1^ s^*−*1^ from transient electroluminescence measurement of hole only device^43^. It is well known that values of mobility measured by different techniques disagree in organic semiconductors^38,44^. Most importantly, results in Table 1 show that the mobility of charger carriers in the printed OLEDs is roughly the same as the mobility in the spin coated OLEDs. Therefore, the adaptation of printing technique for the fabrication of Super Yellow films has no significant effect on the mobility of the charge carriers.

Figure 7c presents the Poole-Frenkel plot (µ_eff_ α ${e}^{{\beta }_{eff}\sqrt{E}}$) of the effective mobility as a function of square root of average electric field (E^1/2^), where, electric field is E = (V−V_bi_) d^*−*1^. *β*_*eff*_ is the Poole-Frenkel coefficient, which indicates the degree of disorder in the film. We found that the effective carrier mobility decreases with increasing average electric field. Similar kind of field dependent mobility is observed elsewhere for OLEDs^41,45^. This kind of mobility dependency results in a negative Poole-Frenkel coefficient. The effective Poole-Frenkel coefficient in spin coated and inkjet printed film is −5.4 × 10^*−*4^ (cmV^*−*1^)^1/2^ and −5.7 × 10^*−*4^ (cmV^*−*1^)^1/2^ respectively, see Supplementary Table S6.

## Conclusions

We demonstrated that the inkjet printing technique can be successfully adopted for fabrication of light emitting layers based on the well-known light emitting polymer- Super Yellow. We formulated the stable ink by dissolving Super Yellow in a mixture of two non-chlorinated solvents: toluene (low boiling point) and tetralin (high boiling point). Such solvents are safe for the elements of printing heads in the most common types of inkjet printing machines. The ink showed excellent wetting on PEDOT:PSS surface with contact angle of less than 8°. We found that parameters, such as the concentration of polymer, the printing resolution and the temperature strongly affect the quality of printed films. After optimizing these parameters, we demonstrated the viability of inkjet printing of high quality Super Yellow thin films with thicknesses from 30 nm to 95 nm. AFM characterization of printed films showed smooth surface with root mean square roughness of 0.55 nm and absence of defects such as pinholes and cracks. The UV-Vis and PL measurements revealed that the optical properties of printed films showed small or negligible changes of their optical properties, in comparison to spin-coated reference films. Transient electroluminescence measurements of effective mobility demonstrated very similar transport properties of inkjet printed and spin-coated films, what might be attributed to similar thin film morphology in both cases. Printed films showed the effective mobility of 2.8 × 10^*−*5^ to 4.1 × 10^*−*5^ cm^2^ V^*−*1^s^*−*1^. Spin-coated films were characterized by the effective mobility of 2.8 × 10^*−*5^ to 4.1 × 10^*−*5^ cm^2^ V^*−*1^s^*−*1^.

We found that the thickness of printed films between 70 nm and 83 nm is an optimum value for obtaining best performance. The printed OLEDs with 83 nm thick Super Yellow layer have shown the highest maximum current efficiency of 5.6 cd/A. The printed OLEDs with 70 nm thick Super Yellow layer have shown the highest maximum luminescence of 64,000 cd/m^2^. The value of maximum luminescence achieved here exceeds previously reported values for SY OLEDs partially fabricated by printing^22,46^. Comparing with SY OLEDs fabricated by spin-coating, maximum luminescence of our printed devices is the same as of our spin-coated devices, and is state-of-the-art^47^. We believe that with further optimization, maximum luminescence of both printed and spin-coated SY OLEDs can achieve values of approx. 100,000 cd/m^2^, published for the best spin coated SY OLEDs fabricated in controlled atmosphere^48,49^. Our experiment confirms that the inkjet printing in ambient conditions is a viable method to obtain bright Super Yellow OLEDs.

In conclusion, we provide full details of the method of fabrication and the characterization of inkjet printed high performance OLEDs based on Super Yellow polymer. We believe that our work will be helpful in the preparation of inkjet inks with non-chlorinated solvents and in the printing of uniform Super Yellow or similar polymer films for OLED applications.

## Experimental Details

### Materials

Toluene and 1,2,3,4-Tetrahydronaphthalene (Tetralin) were purchased from Sigma Aldrich and used as received. Super Yellow (Mw = 1.7 × 10^6^ g mol^−1^, PDY-132) was purchased from Merck Ltd., Germany. Poly(3,4-ethylenedioxythiophene):poly(styrenesulfonate) (PEDOT:PSS) was received from Heraeus, Germany (CLEVIOS P VP AI 4083). It was filtered with 0.45 µm pore sized PVDF filter before using for spin coating. Indium-tin-oxide (ITO) coated substrates were purchased from Osilla Ltd., UK and had sheet resistance of 20 Ω/square.

### Instruments

The printing was performed using PIXDRO LP50 inkjet printer manufactured by MEYER BURGER TECHNOLOGY LTD, Thun, Switzerland. The printer is equipped with industrial grade printhead, Spectra S-Class SE-128 AA supplied from Fujifilm Corporation, Japan. Viscosity of the ink was measured using HAAKE Viscotester 7 supplied by Thermo Fisher Scientific, Karlsruhe, Germany. During all experiments, the temperature of the ink was 25 °C. Surface tension of the ink and the contact angle were measured using OCA 15EC Goniometer purchased from DataPhysics Instruments GmbH, Filderstadt, Germany. Film thicknesses were measured using DektakXT stylus profiler supplied by Bruker Ltd., Coventry, UK. Each sample was scanned in, at least, three different places and averaged data was used the final value of the thickness. The roughness was measured using Flex-Axiom nanosurf AFM purchased from Nanosurf GmbH, Langen, Germany. AFM measurements were made in tapping mode. A UV-VIS-NIR spectrometer (Carry 5000) from Varian Inc., Palo Alto, CA, USA was used to record the absorption spectra of polymer in solution and in solid-state. The photoluminescence of Super Yellow in solution and solid-state was measured using FLS980 photoluminescence spectrometer manufactured by Edinburgh Instruments Ltd. Livingston, UK. Samples were excited with an excitation wavelength of 420 nm. Current - voltage characteristics were measured using Keithley 2400 source measure unit and simultaneously the brightness was recorded using spectroradiometer Minolta supplied by Konica Minolta Sensing Americas, Inc, NJ, USA. Electroluminescence was measured using a MicroHR spectrometer and a CCD camera 3500 (Horiba JobinYvon).

### Fabrication of OLEDs

Inkjet printed polymer OLEDs were fabricated on ITO-coated glass substrates. Schematics of the device structure, the energy levels of constituent layers and of the chemical structure of Super Yellow can be found in Supplementary Fig. S1a–c respectively. Fabrication process was as follows. Firstly, the substrates were cleaned with acetone and isopropyl alcohol. Next, the surface of the ITO was cleaned under the oxygen plasma. Then, the PEDOT:PSS solution was spin-coated at a speed of 2000 rpm for 50 sec, and annealed at 200 °C for 10 min. On the top of PEDOT:PSS layer, the Super Yellow film was inkjet printed or spin coated. This was followed by annealing at 100 °C for 30 min. All above fabrication steps were carried out in ambient conditions. Finally, the calcium (20 nm) and the aluminum (100 nm) were evaporated as cathode using thermal evaporator, connected to a glove box system. In the final step, devices were encapsulated inside a glove box before electrical characterization.

### Transient electroluminescence setup

Schematic of transient electroluminescence setup is shown in Supplementary Fig. S2. A pulse generator was used to provide input voltage pulse with a frequency of 10 kHz to OLEDs. Amplified photodiode (PDA36A, Thorlabs Inc., New Jersey, USA.) was used to detect the light emitted by the OLEDs. Digital oscilloscope (PicoScope 5243 A, PicoTechnology) was used to simultaneously record the applied input voltage pulse, and the signal from the photodiode.

## Supplementary information

Supplementary file
