# Supplementary material for: Inkjet Printing of Super Yellow: Ink Formulation, Film Optimization, OLEDs Fabrication, and Transient Electroluminescence
Source: Sci Rep. 2019 Jun 11;9:8493. doi: 10.1038/s41598-019-44824-w (PMC6560214; doi:10.1038/s41598-019-44824-w)
Supplement: Supplementary file 1 — Supplementary file [file 41598_2019_44824_MOESM1_ESM.docx]

**Inkjet Printing of Super Yellow: Ink Formulation, Film Optimization, OLEDs Fabrication, and Transient Electroluminescence**

**Supplementary Information**

Amruth C ^1^, Marek Zdzislaw Szymanski ^2,3^, Beata Luszczynska ^1^, and Jacek Ulanski ^1^

^1^Department of Molecular Physics, Lodz University of Technology, 90-924, Lodz, Poland

^2^ Department of Engineering and Chemical Sciences, Karlstad University, SE-651 88 Karlstad, Sweden

^3^ School of Science and Technology, Örebro University, SE-701 82, Örebro, Sweden

**Corresponding author:** Beata Luszczynska, [beata.luszczynska@p.lodz.pl](mailto:beata.luszczynska@p.lodz.pl), Tel.: +48 426313216

[amruth.c@p.lodz.pl](mailto:amruth.c@p.lodz.pl) (A.C), [marek@marekszymanski.com](mailto:marek@marekszymanski.com) (M.S.), beata.luszczynska@p.lodz.pl (B.L.), jacek.ulanski@p.lodz.pl (J.U.)


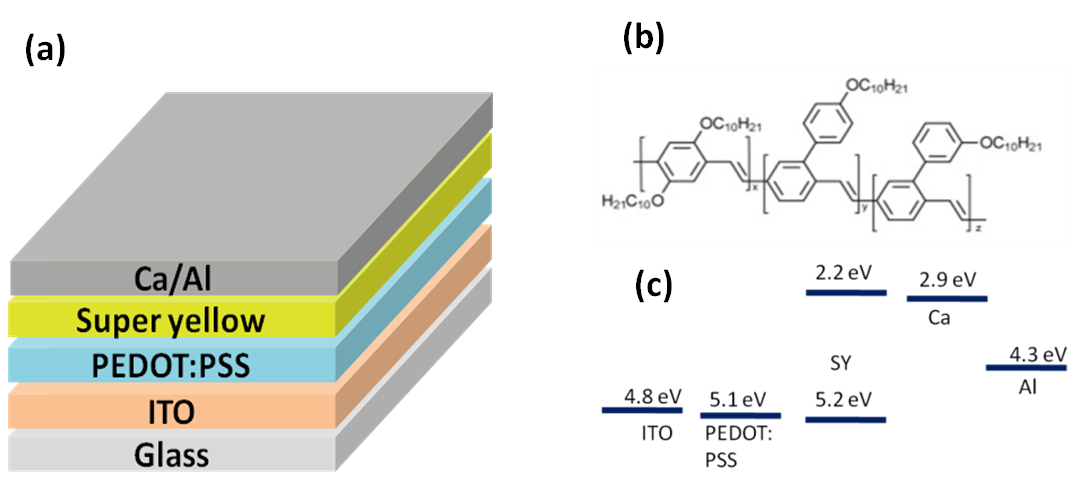


**Figure S1** (a) Schematic diagram of OLED structure (b) chemical structure of Super Yellow (SY) (c) energy level diagram of constituent layers of OLED.

**
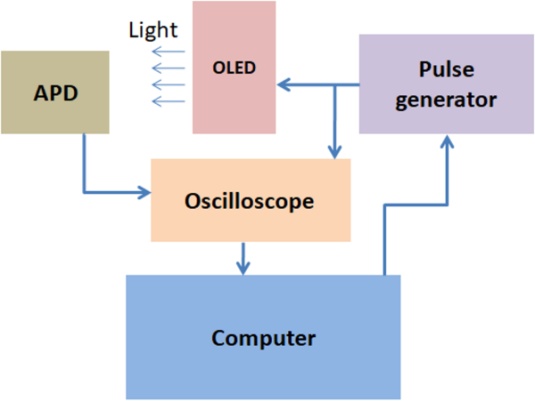
**

**Figure S2** Schematic of experimental set-up for electroluminescence transient measurement. APD: Amplified photodiode (PDA36A) OLED

**Table S1** Physical properties of Super Yellow ink formulations.

| Super Yellow Ink Composition | Viscosity at 25 ^o^C (cP) | Surface Tension (dyne/cm) | Density (g/cm^3^) | Z number |
| --- | --- | --- | --- | --- |
| 0.5 mg/mL in Toluene  (SY INK-1) | 2.5 | 28 | 0.968 | 11.3 |
| 2 mg/mL in Toluene/Tertralin (75:25)  (SY INK-2) | 7 | 30 | 0.988 | 4.6 |
| 3 mg/mL in Toluene/Tertralin (75:25)  (SY INK-3) | 9.1 | 30 | 0.988 | 3.5 |


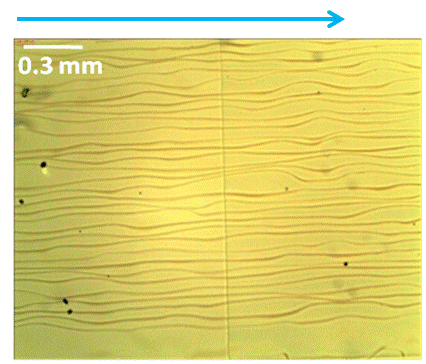


**Figure S3** Optical image of inkjet printed film with SY INK-1. Blue arrow represents the printing direction.


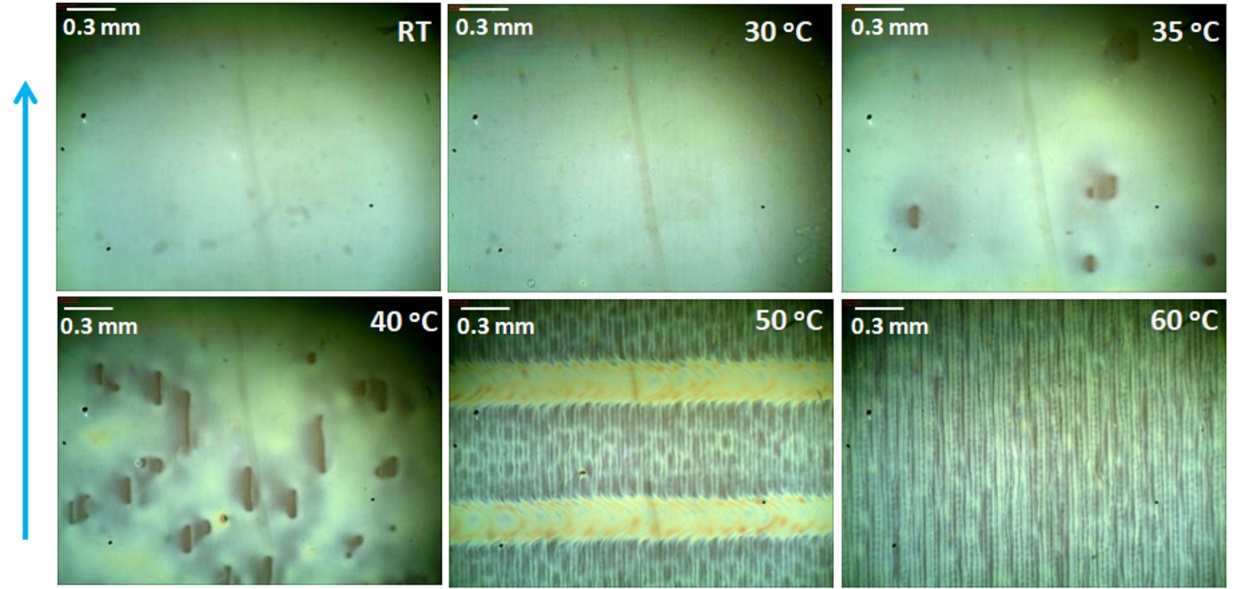


**Figure S4** Optical images of SY films printed on substrates at different temperatures: room temperature (23 °C), 30 ^o^C, 35 ^o^C, 40 ^o^C, 50 ^o^C and 60 ^o^C. SY INK-3 formulation is used, and printing resolution of 700 dpi is used for all temperatures. Blue arrow represents the printing direction.

**
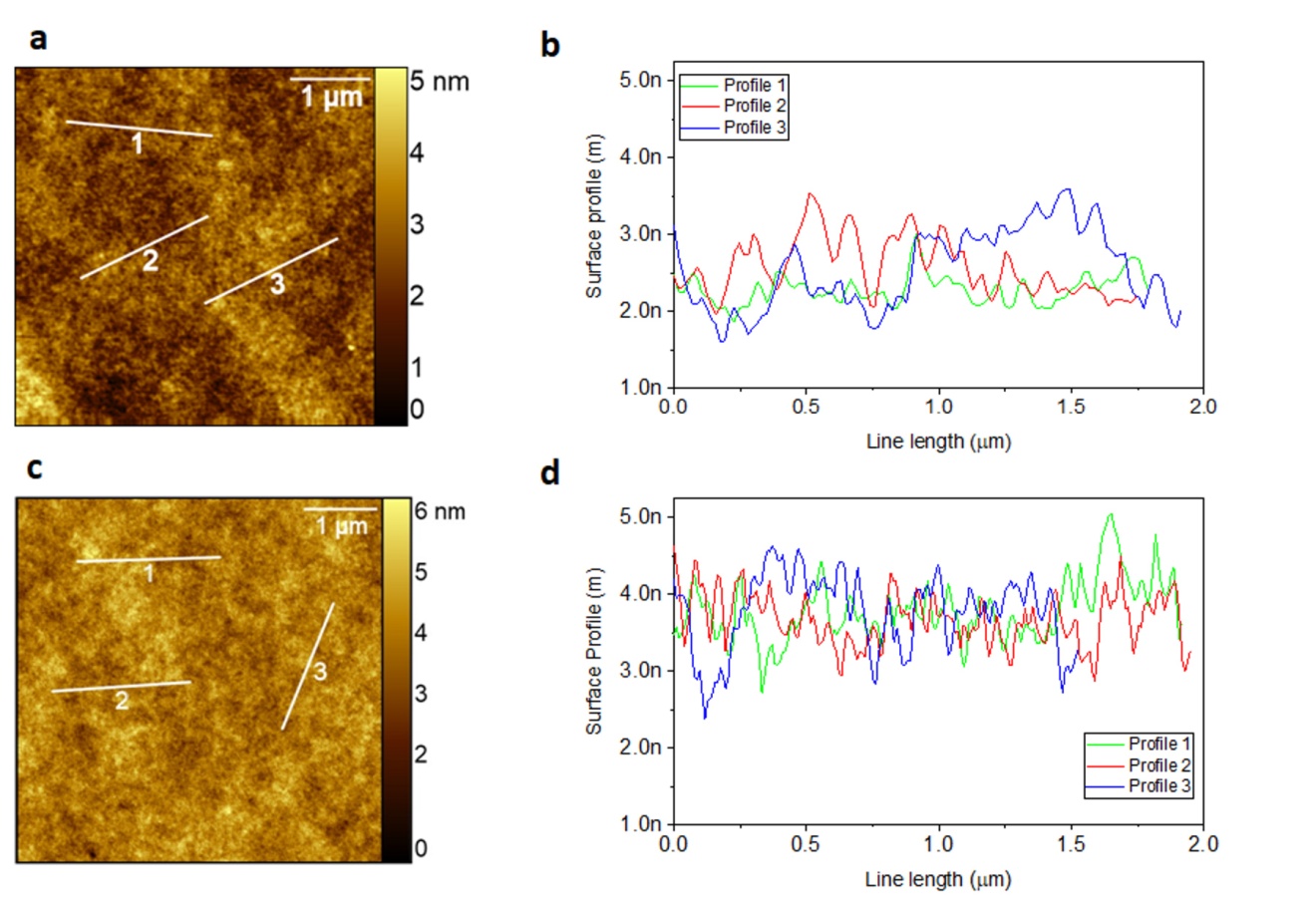
**

**Figure S5** AFM height image (a) and surface profiles taken along three lines (b) of a Spin coated SY film. (c) and (d) are corresponding measurements for inkjet printed SY film.


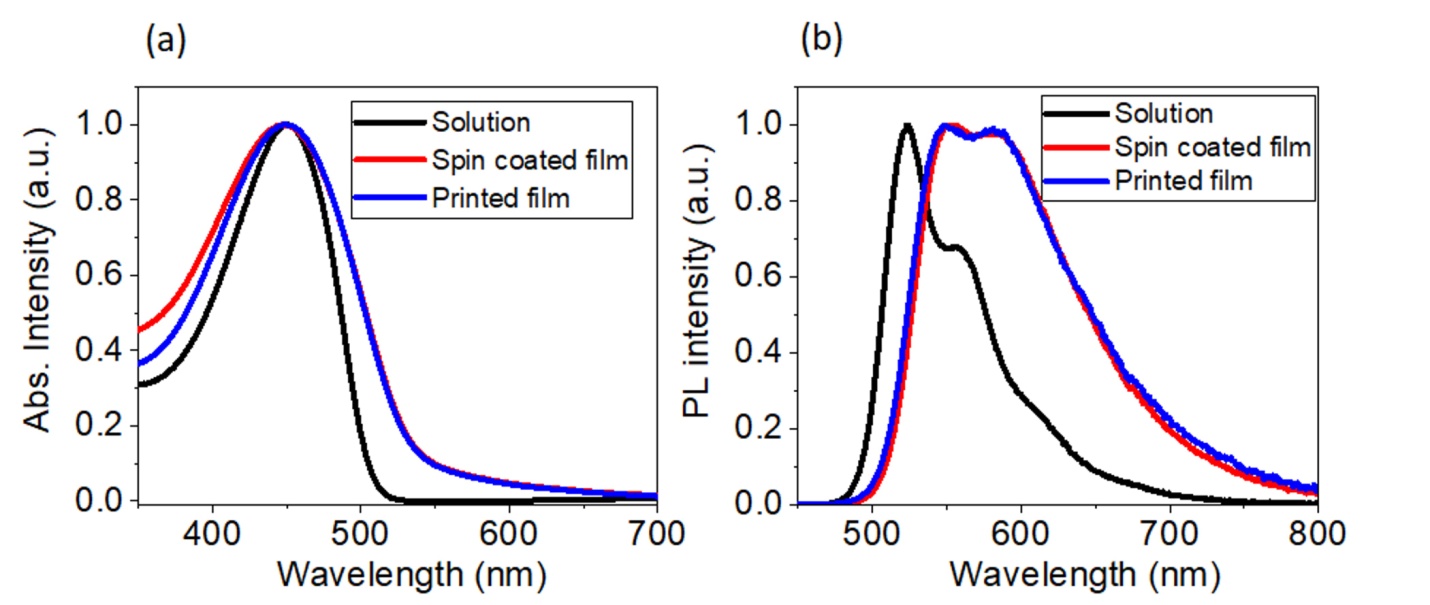


**Figure S6** Normalized UV-visible (a) and photoluminescence (b) spectra of SY in solution, printed thin film, and spin coated thin film.

**
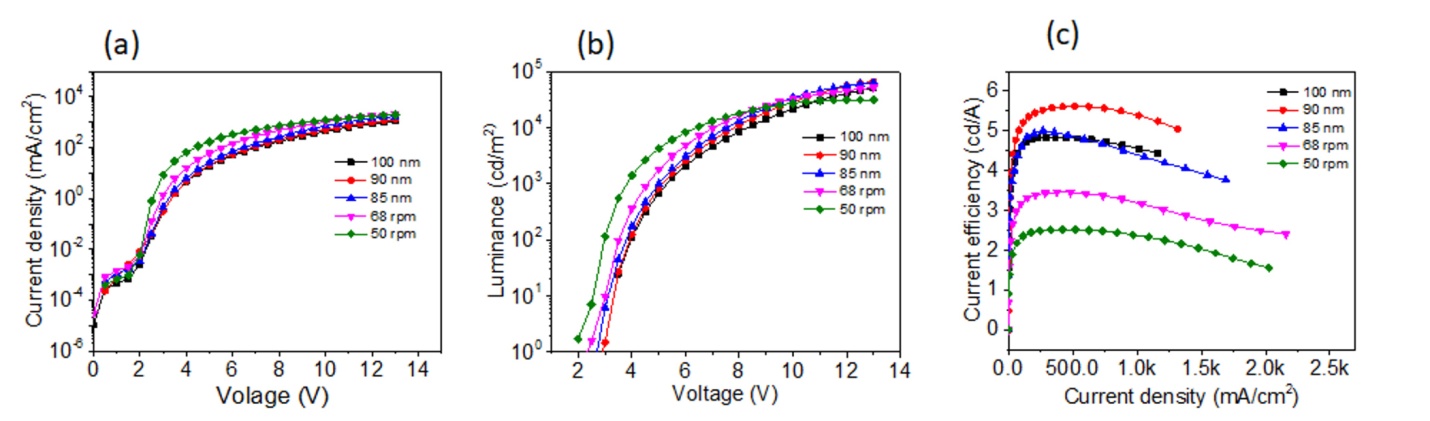
**

**Figure S7:** Current density - voltage characteristics (a), luminance - voltage characteristics (b) and current efficiency - current density characteristics (c) of spin coated OLEDs.

**Table S2** Thickness of Super Yellow films as a function of printing resolution.

| Resolution (dpi) | 500 | 600 | 700 | 800 | 900 |
| --- | --- | --- | --- | --- | --- |
| Thickness (nm) | 33 | 55 | 72 | 83 | 95 |

**Table S3** Turn on voltage, maximum luminance and current efficiency of inkjet printed OLEDs for various thicknesses of Super Yellow layer.

| **Thickness (nm)** | **Turn-on Voltage (V)** | **Max. Luminance (cd/m^2^)** | **Current Efficiency (cd/A)** |
| --- | --- | --- | --- |
| 33 | 2.5 | 14600 | 0.62 |
| 55 | 2.5 | 28000 | 1.4 |
| 72 | 2.5 | 64800 | 4.5 |
| 83 | 2.5 | 45000 | 5.6 |
| 95 | 3 | 43000 | 3.7 |

**Table S4** Thickness of Super Yellow films as a function of spin coating speed.

| **Spin Speed (rpm)** | **1000** | **1200** | **1500** | **2000** | **3000** | **5000** |
| --- | --- | --- | --- | --- | --- | --- |
| **Thickness (nm)** | 100 | 90 | 80 | 68 | 50 | 38 |

**Table S5** Turn on voltage, maximum luminance and current efficiency of spin coated OLEDs for various thicknesses of Super Yellow layer.

| Thickness (nm) | Turn-on Voltage (V) | Max. Luminance (cd/m^2^) | Current Efficiency (cd/A) |
| --- | --- | --- | --- |
| 100 | 3.5 | 51000 | 4.8 |
| 90 | 3 | 66000 | 5.6 |
| 85 | 2.5 | 63000 | 5 |
| 65 | 2.5 | 52000 | 3.4 |
| 50 | 2.5 | 31000 | 2.5 |
| 38 | 2.5 | 29000 | 1.9 |

**Table S6** Effective mobility (µ_eff_) and Poole-Frenkel coefficient (β_eff_) extracted from Poole-Frenkel plot for both spin coated and inkjet printed SY films.

| Devices | µ_eff_ (cm^2^ V^-1^s^-1^) | β_eff_ (cmV^-1^)^1/2^ |
| --- | --- | --- |
| Spin Coated OLEDs | 3.2 × 10^-5^ to 4.3 x 10^-5^ | -5.4 × 10^-4^ |
| Printed OLEDs | 2.8 × 10^-5^ to 4.1 x 10^-5^ | -5.7 × 10^-4^ |
